# Comparative functional exercise capacity of patients with type 2-diabetes and healthy controls: a case control study

**DOI:** 10.11604/pamj.2014.19.257.4798

**Published:** 2014-11-07

**Authors:** Taofeek Oluwole Awotidebe, Rufus Adesoji Adedoyin, Abraar Olalakan Yusuf, Chidozie Emmanuel Mbada, Rose Opiyo, Frasier Chidyaonga Maseko

**Affiliations:** 1 Department of Medical Rehabilitation, College of Health Sciences, Obafemi Awolowo University, Ile - Ife, Nigeria; 2 Department of Nutrition, School of Public Health, University of Nairobi, Nairobi, Kenya; 3 Department of Community Health, College of Medicine, Lilongwe, University of Malawi

**Keywords:** Functional, exercise capacity, hand grip strength, type 2 diabetes

## Abstract

**Introduction:**

Functional Exercise Capacity (FEC) is a valid measure of physical fitness in health and disease. However, there is paucity of studies on FEC in African patients with Type-2 Diabetes (T2D). This study compared FEC between patients with T2D and healthy controls.

**Methods:**

Thirty five patients with T2D (18 men, 17 women) and 35 (16 men, 19 women) age-sex matched healthy controls participated in this case-control study. Anthropometric and demographic characteristics and cardiovascular parameters were measured following standard procedures. A glucometer was used to determine the Fasting Blood Glucose (FBG) level following at least 8 hours of overnight fasting. FEC was assessed using the Six-Minute Walk Test (6MWT) while Hand Grip Strength (HGS) test was measured with an electronic dynamometer. Data were analyzed using descriptive and inferential statistics. Alpha level was set at p< 0.05.

**Results:**

Patients with T2D and controls were similar in age (p > 0.05). There were significant differences in the distance covered during 6MWT between patients and controls (t= 0.329; p =0.03), exercise capacity (t = 0.329; p = 0.03), FBG (t = 7.403; p = 0.001), systolic and diastolic blood pressure (t = 12.56; p = 0.001 and t = 27.23; p = 0.001) respectively. There were significant inverse relationships between 6MWD and Body mass index (r = -0.39; p = 0.02) and FBS(r = -0.51; p = 0.02) in patients with type-2 respectively. No significant association was found between exercise capacity and HGS (p > 0.05).

**Conclusion:**

Patients with type-2 diabetes demonstrated lower functional exercise capacity than healthy controls. High body mass index and fasting blood glucose were significantly associated with lower functional exercise capacity.

## Introduction

The prevalence of Type-2 Diabetes (T2D) is on the rise globally, now occurring in about 6% of the world's adult population [1, 2]. Sub-Sahara Africa (SSA) is also witnessing a significant increase in T2D alongside other non-communicable diseases [3]. Hall et al, [4], reported that the prevalence of T2D in some selected western African countries, including Nigeria and Ghana, to be up to 7%, while in South Africa and Uganda prevalence is reported to be more than 10%. Several predisposing factors have been attributed to the increase in the prevalence of T2D including rapid urbanization, poor eating habits, physical inactivity and obesity [5, 6]. It has been proposed that high prevalence of T2D complications will impose a huge burden on the healthcare system in SSA if urgent interventions are not taken [7]. There is substantial evidence that individuals with T2D are more vulnerable to various short and long term complications associated with premature death [8]. This vulnerability has been attributed to insidious onset [9], delay in diagnosis and late presentation [10], poor glycemic control [11] and late recognition of symptoms and economic cost of care in low resource nations, especially SSA countries [12, 13]. The culture of regular medical check-up is lacking in developing nations, hence many cases of undiagnosed T2D are undiagnosed. Experts have identified limited availability of diagnostic testing tools and non-standardized diagnostic criteria leading to poor treatment plan in SSA as problems [14, 15]. In the management of T2D, pharmacologic therapies have been proved to be effective in the control of blood sugar and reduction in the common complications [16]. In spite of this, many patients still remain burdened with reduced physical functioning, poor exercise tolerance, disability and vulnerable to cardiovascular risks [17]. However, exercise training has been reported to be effective as an adjunct therapy in improving exercise tolerance and physical fitness [18]. One of the ways of improving exercise prescription and fitness in patients with T2D is to establish evidence on physical fitness measures such as functional exercise capacity, muscular strength and cardiovascular endurance tests.

Assessment of functional exercise capacity is usually a laboratory-base test and it is complex, time consuming and expensive. Devices such as treadmill or bicycle ergometry test with Douglas bag and gas analyzer are frequently used for graded exercise stress test [19]. In poorer settings these assessments are usually unaffordable [20], and appropriate expertise to conduct these tests is lacking [21]. Hence, an assessment technique that is simple to administer, cost-effective, well tolerated and not time consuming will be an added advantage for countries with limited resources for both patients with T2D and their clinicians. Adedoyin et al, [22] used a hand grip strength test to assess functional exercise capacity in patients with chronic obstructive pulmonary disease [20] and a 6 Minute Walk Test (6MWT) to determine exercise capacity in patients with chronic heart failure. Unfortunately, studies comparing functional exercise capacity of African patients with T2D with healthy controls are scanty. Measuring the functional exercise capacity in patients with T2D could serve as standard basis for exercise prescription, training and improvement in clinical outcomes. Therefore, this study assessed and compared functional exercise capacity of patients with T2D and age-sex matched apparently healthy controls.

## Methods

This case-control study recruited thirty five patients diagnosed with T2D from the Endocrine Clinic, Obafemi Awolowo University Teaching Hospitals Complex (OAUTHC), Ile - Ife and 35 apparently healthy adults; age and sex matched controls from patient relatives and hospital workers using purposive sampling technique. Eligibility for inclusion in the study were patients whose ages range between 35-70 years and attending the Endocrine Clinic of the OAUTHC having been confirmed of T2D by clinical diagnosis. Apparently healthy age and sex-matched control with no self-reported previous history or diagnosis of T2D were also included. Individuals with associated musculoskeletal disorders such severe arthritis of the knee or hip joint, cardiovascular disease affecting the lower limb such as peripheral vascular disease or neurological condition such as peripheral neuropathy were excluded from the study.

### Procedures

Prior to the conduct of the study, the purpose was explained to the participants who met inclusion criteria while informed consent was obtained. The physical characteristics of the participants including height and weight, waist and hip circumferences were measured and the body mass index (BMI) was calculated. Cardiovascular parameters of systolic, diastolic blood pressure and heart rate were measured using standard procedures. Demographic data including level of education, type of occupation and monthly income were also obtained.

### Biochemical analysis

#### Fasting Blood Glucose (FBG)

The day prior to the assessment of FBG, participants were informed not to eat any food or drink for a minimum of 8 hours before their appointment. All measurements were taken between 7.00 am to 9.00 am. A One Touch Lifescan Glucometer; Johnson-Johnson Company Product, Inc., Milpitas, CA 95035 was used to test the glucose level using a standard procedure [23]. A fasting Blood Glucose greater than 5.6mmol/L confirms the presence of diabetes.

### Assessment of functional exercise capacity

#### Six-Minute Walk Test

The Six-Minute Walk Test (6-MWT) was performed on a 20 meter level corridor without any obstructing object using American Thoracic Society [24] guidelines. Participants were allowed to rest for period of 10 minutes in sitting position before the commencement of the exercise test. Participants were instructed to walk from the starting point to the end at their own selected pace while attempting to cover as much ground as possible in six minutes [20]. Encouragement was provided every 30 seconds or more in a standardized manner by saying: “You are doing well” or “Keep up the good work”. Patients were then asked to rate his or her exertion level using the modified Borg's scale “0” represents “no discomfort”; “5” represents “moderate”; “10” represents extreme discomfort”. The maximum oxygen consumption (VO2 max) was calculated from the American College of Sport Medicine [25] prediction equation while Metabolic Equivalent (MET) was derived from VO2 max by dividing it with 3.5. The six minute walk work (6MWW) was calculated from the product of 6MWD and the body weight of the participant [26].

#### Hand Grip Strength

Each participant's hand grip strength was assessed with the aid of an electronic Camry hand grip dynamometer (Model EH 101, made in Taiwan) based on the recommendation of the American Society of Hand Therapist [27]. The participant sat on a straight-back and armless chair of standard height with participant's test arm was held at 90o elbow flexion position and the forearm in neutral position without radio-ulnar deviation. The hand was positioned parallel to the forearm holding the dynamometer. Participant was instructed to squeeze maximally and hold until the reading is taken. Two measurements were taken for each upper extremity with a 2-minutes rest interval; the average was recorded in kilogram-force (Kgf) as grip strength value. For standardization, the dynamometer was set at the second handle position. No verbal encouragement was given [22]. Ethics and Research Committee of the Institute of Public Health (IPH), Obafemi Awolowo University, Ile - Ife gave approval for the study's protocol. The Head of Endocrine Unit of the Obafemi Awolowo University Teaching Hospitals Complex, Ile-Ife, Nigeria gave permission to conduct the study.

#### Data analysis

Descriptive statistics of frequency, percentage, mean and standard deviation were used to summarize data. Chi square test was used to determine the associations of age and socio-demographic variables between patients and healthy controls. Independent t-test was used to compare physical characteristics, grip strength, exercise capacity and cardiovascular parameters of patients with T2D and healthy controls. Pearson Product Moment Correlation was used to determine the relationship between physical characteristics, FBG, hand grip strength (dominant) and functional exercise capacity of patients with T2D and healthy controls. Alpha level was set at 0.05 of significance. Data analysis was performed using STATA-SE version 11.0 software (STATA Corp, Texas, USA).

## Results

There was no statistically significant difference in main demographic variables between the case and control groups (Table 1). There were significant differences between the cardiovascular parameters of patients with T2D and healthy controls in six minute walk distance (t = 0.33; p = 0.03), exercise capacity (t = 0.33; p = 0.03), systolic and diastolic blood pressure (t = 12.56; p = 0.01 and t= 27.23; p = 0.01) respectively (Table 2). Table 3 showed the result of Pearson moment correlation with moderate significant inverse relationships between 6MWD and Body mass index (r= -0.39; p = 0.02) in patients with T2D but not with healthy control. No significant correlation was found between BMI and HGS in both groups (r = 0.18; p = 0.30 and r = 0.01; p = 0.98 respectively. There was a significant correlation between exercise capacity and FBG (r =-0.51; p = 0.02) in patients with T2D but there was no significant correlation in healthy controls. Significant correlations were found between exercise capacity and 6MWW (r = 0.62; p = 0.001) in patients with type-2 and as well as healthy control (r= 0.72; p = 0.001) respectively. No significant correlation was found in the HGS in both groups (p > 0.05).

**Table 1:** Comparison of physical and socio-demographic characteristics of patients with type-2 and healthy controls

Variable	Patients (n = 35) Mean ±S.D	Controls (n = 35) Mean ±S.D	t-cal	p-value
**Physical Characteristics**				
Age (years)	58.0±8.8	58.1±8.9	0.001	1.00
Weight (Kg)	71.5±13.7	67.4±8.9	0.004	0.14
Height (m)	1.7± 0.1	1.7±0.1	0.284	0.18
BMI (kg/m2)	25.8±4.7	23.1±2.1	0.001	0.04*
WC (cm)	83.5±12.6	72.9±7.3	5.130	0.01*
WHR	0.9 ± 0.1	0.8 ± 0.1	-3.600	0.01*
FBG (mmol/L)	5.7 ± 0.7	4.7 ± 0.6	7.403	0.01*
**Socio-demographics**	n (%)	n (%)	c2	p-value
Gender				
Male	18(51.4)	16(45.7)	0.23	0.81
Female	17(48.6)	19(54.3)		
**Occupation**				
Civil servant	9(25.3)	7(20.0)	0.55	0.76
Retiree	7(20.0)	6(17.1)		
Trading/Artisan	19(54.7)	22(62.9)		
**Education level**				
Primary	15(42.8)	12(34.3)	3.17	0.21
Secondary	11(31.4)	18(51.4)		
Tertiary	9(25.3)	5(14.3)		
**Monthly Income**				
< #50,000	3(8.6)	4(11.4)	3.83	0.28
#50,000 - #75,000	11(31.4)	10(28.6)		
> # 76 - #100,000	16(45.7)	10(28.6)		
> #100,000	5(14.3)	11(31.4)		

* Significant at p< 0.05

Keys: BMI, Body Mass Index; WC, Waist Circumference; HC, Hip Circumference; WHR, Waist to Hip Ratio; FBG, Fasting Blood Glucose

**Table 2:** Comparison of cardiovascular parameters and functional capacity test between patients with type 2 diabetes and healthy controls

Variable	Patient Group X ± S.D	Control Group X ± S.D	t-cal	p-value
**Blood pressure**				
Systolic (mmHg)	135.0±20.2	119.0±7.9	12.56	0.001*
Diastolic (mmHg)	79.7±11.4	74.6±8.3	27.23	0.001*
Heart rate (beat/sec)	75.0±7.4	72.9±7.4	29.78	0.001*
**Exercise capacity**				
6MWD (m)	403.0±88.3	447.0±7.7	0.329	0.030*
VO_2_max (mL/kg/min)	10.2±1.5	10.9±1.3	0.329	0.030*
6MWW X10^3^ (Kg.m)	28.0 ± 6.6	29.9 ± 6.0	0.983	0.230
RPE	3.4±1.2	2.2±0.7	5.495	0.001*
**Hand grip strength**				
Dominant (Kgf)	27.0±5.8	29.3±4.9	0.320	0.080
Non-dominant (Kgf)	25.2±5.0	26.9±4.1	0.220	0.120

* Significant at p< 0.05

Keys: 6MWD - 6-Minute Walk Distance; VO_2_Max - Maximum Oxygen Consumption; 6MWW; 6-Minute Walk Work; RPE, Rate of Perceived Exertion

**Table 3:** Relationship between age, anthropometric characteristics, cardiovascular, fasting blood glucose and each of functional capacity variables in patients with type-2 diabetes and apparently healthy controls

	Patients		Control	
	6MWD	HGSD	6MWD	HGSD
Variables	r(p)	r(p)	r(p)	r(p)
Age (years)	-0.32(0.06)	-0.25(0.15)	-0.32(0.06)	-0.25(0.16)
BMI (kg/m^2^)	-0.39(0.02)*	0.18(0.30)	-0.16(0.36)	0.01(0.98)
WC (cm)	-0.08(0.63)	0.18(0.31)	0.03(0.86)	0.21(0.22)
WHR	-0.06(0.71)	-0.23(0.19)	-0.13(0.47)	0.09(0.59)
SBP (mmHg)	-0.01(0.95)	0.06(0.73)	-0.29(0.09)	-0.01(0.94)
DBP (mmHg)	-0.18(0.29)	0.16(0.37)	0.04(0.82)	0.11(0.54)
FBG (mmol/L)	-0.51(0.02)*	0.01(0.96)	-0.21(0.24)	0.32(0.06)
6MWW	0.62(0.001) **	0.20(0.25)	0.72(0.001) **	-0.08(0.66)

* Significant at p< 0.05

** Significant at p< 0.001

Keys: 6MWD, 6-Minute Walk Distance; HGSD, Dominant Hand Grip Strength; BMI, Body Mass Index; WC, Waist Circumference; WHR, Waist to Hip Ratio; FBG, Fasting Blood Glucose; SBP, Systolic Blood Pressure; DBP, Diastolic Blood Pressure

## Discussion

This study was designed to investigate the functional exercise capacity of patients with T2D and healthy controls. Findings from this study show that patients with T2D demonstrated lower exercise capacity compared with healthy controls. This is in agreement with the findings of previous studies which found that lower exercise capacity is common in patients with T2D [28, 29]. The reduction in exercise capacity of patients with T2D may be linked to poor glucose metabolism. The transporter protein GLUT4 expression at the plasma membrane is related to fibre volume in human skeletal muscle fibres [30]. Poor glycemic control in patients with T2D has been associated with increased stiffness of large conduit vessels. The compliance of the aorta plays significant role in modulating coronary artery blood flow which has important consequences for myocardial work capacity and, therefore, leading to reduced exercise capacity [31].

There is growing body of evidence to suggest that body's capability to utilize oxygen in patients with T2D is compromised due to poor blood perfusion of muscles at cellular level and consequently reduction in functional exercise capacity [32]. This has been suggested as one of the factors limiting the ability to achieve peak performance during maximal exercise. Although, the 6MWT is a submaximal test, quite a large number of patients with T2D do not meet minimal performance during sub-maximal test [33]. Thus, the distance covered during 6 minute walk has become a well-established predictor of cardiovascular risk and all-cause mortality in patients with T2D. Carter et al, [26], however, observed that body weight was not taken into account during 6MWT which is considered to be a potential factor during walking performance. Hence, the Six Minute Walk Work (6MWW) which is the product of body weight and distance covered during six minute walk has been advocated to assess functional exercise capacity [34]. Weight control is an important lifestyle modification intervention in diabetes control which may be a useful indicator for improvement during assessment.

Exercise prescription is usually based on heart rate and other cardiovascular parameters in order to determine exercise safety and progression. Findings from this study showed that there were significant differences between cardiovascular parameters of patients with T2D and healthy control. This is in line with the findings of previous studies that reported impaired cardiovascular function in T2D [28, 31]. Furthermore, abnormal cardiac autonomic function has been associated with poor cardiac output responses to exercise in diabetes, hence exercise prescription should be carefully planned among this population. Previous studies on physical activity level among patients with T2D have reported to be below optimal level [35, 36]. Thus, weaker muscles as a result of inactivity tend to be smaller and have potential for poor glucose uptake and hyperglycemia. The effect of chronic diseases with attending co-morbidities could impact negatively on the functioning of the cardiovascular system. The heart in a compensatory manner may attempt to relieve the burden of the underlying pathologies, hence resulting in a decline in the efficient distribution of blood through the body tissues [32].

Studies on Hand Grip Strength (HGS) in patients with T2D have been reported to be poor [37, 38], but our findings showed that HGS of patients with T2D was not significantly different from healthy controls. This may be attributed to good blood glucose control among the study population leading to optimal muscular strength. A study by Wander et al, [39] reported that the important biomarker of survival in a diabetic population included hand grip strength, described as a measure of total body strength which is significantly associated with physical performance [40]. Although several factors such as advancing age, mood, time of the day and anthropometric characteristics have been reported as possible influences that may affect HGS [41], progressive muscle weakness and limited joint mobility and other pathological manifestations may contribute to poor HGS [42, 43]. Nonetheless, progressive resistance exercise training has been reported to be effective in improving muscular strength in patients with T2D [44].

The potential limitation of the present study could be a number of existing underlying pathology such as cardiovascular disease, previous history of physical inactivity and unhealthy lifestyle thus contributing to low functional exercise capacity. Furthermore, there are few studies on functional exercise capacity in African patients with T2D which makes it difficult for detailed comparison. It is also possible that the cases and controls were too closely matched as a large number of the controls were from the same family population rather than the general population. Correlation between exercise capacity and FBG, although moderate, may have accounted for low functional exercise capacity of these people.

## Conclusion

Patients with type-2 diabetes demonstrated lower functional exercise capacity than healthy controls. High body mass index and fasting blood glucose were significantly associated with lower functional exercise capacity. Optimal control of glucose and body weight may help to maintain adequate functional exercise capacity in patients with type-2 diabetes.

## References

[1] Meetoo D, McGovern P, Safadi R (2007). “An epidemiological overview of diabetes across the world”. Br J Nurs..

[2] Melmed S, Polonsky KS, Larsen PR, Kronenberg HM (2011). Williams textbook of endocrinology.

[3] BeLue R, Okoror TA, Iwelunmor J, Taylor KD, Degboe AN, Agyemang C, Ogedegbe G (2009). An overview of cardiovascular risk factor burden in sub-Saharan African countries: a socio-cultural perspective. BMC Globalization and Health.

[4] Hall V, Thomsen RW, Henriksen O, Lohse N (2011). Diabetes in Sub Saharan Africa 1999-2011: Epidemiology and public health implications; a systematic review. BMC Public Health.

[5] Carulli L, Rondinella S, Lombardini S, Canedi I, Loria P, Carulli N (2005). “Review article: diabetes, genetics and ethnicity”. Aliment Pharmacol Ther..

[6] Adegoke OA, Adedoyin RA, Balogun MO, Adebayo RA, Bisiriyu LA, Salawu AA (2010). Prevalence of metabolic syndrome in a rural community in Nigeria. Metab Syndr Relat Disord..

[7] Alberti KG, Zimmet P, Shaw J (2007). International Diabetes Federation: a consensus on Type-2 diabetes prevention. Diabet Med..

[8] Saydah SH, Eberhardt MS, Loria CM, Brancat FL (2002). Age and the burden of death attributable to diabetes in the United States. Am J Epidemiol..

[9] Mbanya JC, Sobngwi E (2003). Diabetes microvascular and macrovascular disease in Africa. Eur J Prev Cardiol..

[10] Nyenwe EA, Odia OJ, Ihekwaba AE, Ojule A, Babatunde S (2003). Type-2 diabetes in adults Nigeria; a study of its prevalence and the risk factors in Port Harcourt, Nigeria. Diabetes Res Clin Pract..

[11] Amoah AGB, Owusu SK, Adjei S (2002). Diabetes in Ghana: a community based prevalence study in Greater Accra. Diabetes Res Clin Pract..

[12] Aubeni RE, Herman WH (1998). Global burden of diabetes 1995-2005: prevalence, provisional estimates and projections. Diabetes Care..

[13] Sen K, Bonita R (2000). Global health status; two step forward one step backward. Lancet..

[14] Beran D, Yudkin JS (2006). Diabetes care in sub-Saharan Africa. Lancet..

[15] Beran D, Yudkin JS (2010). Looking beyond the issue of access to insulin: What is needed for proper diabetes care in resource poor settings. Diabetes Res Clin Pract..

[16] Ripsin CM, Kang H, Urban RJ (2009). “Management of blood glucose in type-2 diabetes mellitus”. Am Fam Physician..

[17] Sharma AM (2002). Adipose tissue: a mediator of cardiovascular risk. Int J Obes Relat Metab Disord..

[18] Warburton DER, Nicol CW, Bredin SSD (2006). Healthy benefits of physical activity: the evidence. CMAJ..

[19] ATS/ACCP (2003). Statement on cardiopulmonary exercise testing. Am J Respir Crit Care Med..

[20] Adedoyin RA, Adeyanju SA, Balogun MO, Akintomide AO, Adebayo RA, Akinwusi PO, Awotidebe TO (2010). Assessment of exercise capacity in African patients with chronic heart failure using six minute- walk. Int J Gen Med..

[21] Santos JJA, Brofman PRS (2008). Six-minute walk test and quality-of-life in heart failure: a correlative study with a Brazilian sample. Insuficiencia Cardiaca..

[22] Adedoyin RA, Awopeju OF, Oyadeyi O, Obaseki DO, Ojo O, Erhabor GE (2011). Comparism of functional capacity of patients with chronic obstructive pulmonary disease and post-tuberculosis lung disorders. Nig J Health Sci..

[23] American Diabetes Association (2012). “Standards of medical care in diabetes -2012.”. Diabetes Care..

[24] American Thoracic Society (2002). Guidelines for six-minute walk test. Am J Respir Crit Care Med..

[25] American College of Sport Medicine American College of Sport Medicine (1980). Guidelines for graded exercise testing and exercise Prescription, Lea and Fabiyer.

[26] Carter R, Holiday DB, Nwazuruba C, Stocks J (2003). 6 minutes’ walk work for assessment of functional capacity in patients with COPD. Chest..

[27] American Society of Hand Therapist American Society of Hand Therapists ASHT, editor (1981). Clinical assessment recommendations.

[28] Verges B, Patois-Verges B, Cohen M (2004). Effects of cardiac rehabilitation on exercise capacity in Type 2 diabetic patients with coronary artery disease. Diabet Med..

[29] Vancampfort D, De Hert M, Sweers K, De Herdt A, Detraux J, Probst M (2013). Diabetes, physical activity participation and exercise capacity in patients with schizophrenia. Psychiatry Clin Neurosci..

[30] De Rekeneire N, Resnick HE, Schwartz AV, Kuller LH, Shorr RI, Kuller LH, Simonsick EM, Vellas B, Harris TB (2003). Diabetes is associated with sub-clinical functional limitation in non-disabled older individuals; the health, aging and body composition study. Diabetes Care..

[31] Fang ZY, Sharman J, Prins JB, Marwick TH (2005). Determinants of exercise capacity in patients with type 2 diabetes. Diabetes Care..

[32] Kingwell BA, Formosa M, Muhlmann M, Bradley SJ, Mcconell GK (2003). Type 2 Diabetic individuals have impaired leg blood flow responses to exercise: role of endothelium-dependent vasodilation. Diabetes Care..

[33] Fang ZY, Prins JB, Marwick TH (2004). Diabetic cardiomyopathy: evidence, mechanisms, and therapeutic implications. Endocr Rev..

[34] Golpe R, Pérez-de-Llano LA, Méndez-Marote L, Veres-Racamonde A (2013). Prognostic significance of distance, work, oxygen saturation and dyspnea during 6-minute walk test in COPD patients. Respiratory Care..

[35] Adeniyi AF, Uloko AE, Sani-Suleiman I (2010). Relationship between the six-minute walk test and correlates of type-2 diabetes; Indication for caution in exercise prescription. AJPARS..

[36] Fagour C, Gonzalez C, Pezzino S, Florenty S, Rosette-Narece M, Gin H, Rigalleau V (2013). Low physical activity in patients with type 2 diabetes: the role of obesity. Diabetes Metab..

[37] Celtinus E, Buyukbese MA, Uzel M, Ekerbicer H, Karaoquz A (2005). Hand grip strength in patients with type 2 diabetes mellitus. Diabetes Res Clin Pract..

[38] Savas S, Köroğlu BK, Koyuncuoğlu HR, Uzar E, Celik H, Tamer NM (2007). The effects of the diabetes related soft tissue hand lesions and the reduced hand strength on functional disability of hand in type 2 diabetic patients. Diabetes Res Clin Pract..

[39] Wander PL, Boyko EJ, Kahn SE (2011). Greater hand grip strength predicts a lower risk of developing type 2 diabetes over 10 years in leaner japanese americans. Diabetes Res Clin Pract..

[40] Adedoyin RA, Ogundapo FA, Mbada CE, Adekanla BA, Johnson OE, Onigbinde AT, Emechete AAI (2009). Reference values for hand grip strength among healthy adults in Nigeria. Hong Kong Physiother..

[41] Sayer AA, Dennison EM, Sydaball HE, Gilbody HJ, Phillips DW, Cooper C (2005). Type 2 diabetes, muscle strength and impaired physical function. Diabetes Care..

[42] Mota M, Panus C, Mota E, Sfredel V, Patrascu A, Vanghelie L (2000-2001). Hand abnormalities of the patients with diabetes mellitus. Rom J Intern Med..

[43] Cagliero E, Apruzzese W, Perlmutter GS, Nathan DM (2002). Musculoskeletal disorders of the hand and shoulder in patients with diabetes mellitus. Am J Med..

[44] Willey KA, Fiatarone Singh MA (2003). Battling insulin resistance in elderly obese people with type 2 diabetes: Bring on the heavy weights. Diabetes Care..

